# *Cc*GSDMEa functions the pore-formation in cytomembrane and the regulation on the secretion of IL-lβ in common carp (*Cyprinus carpio haematopterus*)

**DOI:** 10.3389/fimmu.2022.1110322

**Published:** 2023-01-06

**Authors:** Yanjing Zhao, Jie Zhang, Dan Qiao, Feng Gao, Yanlong Gu, Xinyu Jiang, Lei Zhu, Xianghui Kong

**Affiliations:** Engineering Lab of Henan Province for Aquatic Animal Disease Control, College of Fisheries, Henan Normal University, Xinxiang, Henan, China

**Keywords:** common carp, *Aeromonas hydrophila*, GSDMEa, caspases, gasdermin pore, IL-lβ secretion

## Abstract

GSDME is the only direct executor of caspase-dependent pyroptosis in both canonical and non-canonical inflammasomes known to date in fish, and plays an important role in anti-bacterial infection and inflammatory response. In order to determine the regulation of GSDMEa on antibacterial infection in innate immune response, the *CcGSDMEa* gene in common carp (*Cyprinus carpio haematopterus*) was first identified and characterized, and then its function related to immune defense was investigated. Our results showed that the expressions of *Cc*GSDMEa at the mRNA and protein levels were both significantly increased after *Aeromonas hydrophila* intraperitoneal infection at the early stage than that in the control group. We found that *Cc*GSDMEa could be cleaved by inflammatory caspase (*Cc*Caspase-1b) and apoptotic caspases (*Cc*Caspase-3a/b and *Cc*Caspase-7a/b). Interestingly, only the *Cc*GSDMEa-NT (1-252 aa) displayed bactericidal activity to *Escherichia coli* and could punch holes in the membrane of HEK293T cells, whereas *Cc*GSDMEa-FL (1-532 aa) and *Cc*GSDMEa-CT (257-532 aa) showed no above activity and pore-forming ability. Overexpression of *Cc*GSDMEa increased the secretion of *Cc*IL-1β and the release of LDH, and could reduce the *A. hydrophila* burdens in fish. On the contrary, knockdown of *Cc*GSDMEa reduced the secretion of *Cc*IL-1β and the release of LDH, and could increase the *A. hydrophila* burdens in fish. Taken together, the elevated expression of *Cc*GSDMEa was a positive immune response to *A. hydrophila* challenge in fish. *Cc*GSDMEa could perform the pore-formation in cell membrane and the regulation on the secretion of IL-lβ, and further regulate the bacterial clearance *in vivo*. These results suggested that *Cc*GSDMEa played an important role in immune defense against *A. hydrophila* and could provide a new insight into understanding the immune mechanism to resist pathogen invasion in teleost.

## Introduction

The common carp (*Cyprinus carpio haematopterus*), as an important economic species, is famous for rich nutrition, fast growth rate and long-standing history of breeding, and contributes a lot to freshwater aquaculture (1). The expansion of farming scale and the emergence of intensive rearing bring economic benefits, but also lead to the outbreaks of various diseases at the meantime. Bacterial hemorrhagic septicemia caused by *Aeromonas hydrophila* is one of the main diseases, which is ferocious and causes fish massive die-offs in severe case. *A. hydrophila* is a Gram-negative short rod-shaped bacterium, which is widely distributed in various water and can cause severe diseases in fish, reptiles, amphibians and humans under certain conditions (2). In aquaculture, when environmental factors suddenly change and water quality deteriorates, *A. hydrophila* can infect various fish species, such as Indian major carp, mrigal *Cirrhinus mrigala* (3), rainbow trout *Oncorhynchus mykiss* (4), common carp *C. carpio* (5, 6), crucian carp *Carassius auratus* (7, 8), grass carp *Ctenopharyngodon idellus* (9), zebrafish *Danio rerio* (10), channel catfish *Ictalurus punctatus* (11–13), and tilapia *Oreochromis niloticus* (14), and lead to the outbreaks of bacterial hemorrhagic septicemia. This bacterial septicemia disease affected the quality and safety of the aquatic products, seriously restricted the sustainable development of healthy aquaculture, and resulted in considerable economic losses (2, 15). Hence, it is particularly important to determine the immune response of fish to the *A. hydrophila* infection, and to provide an effective strategy for controlling and preventing this bacterial septicemia.

In fish, innate immunity is considered as the major form of host defense, and it is also the efficient first line to resist invading pathogen (8). After the infection of *A. hydrophila*, the innate immune response is activated to improve the immune ability in teleost, and effectively eliminate pathogenic bacteria. Studies have shown that gasdermin E (GSDME) plays an indispensable role in combatting pathogen invasion through its NT-domain in teleost (16–20). The GSDME is composed of a conserved N-terminal pore-forming domain (N-PFD), a C-terminal auto-inhibitory domain (C-AID), and an intermediate linker region with a caspase hydrolysis site (18, 19, 21, 22). Under the normal condition, GSDME exists in inactive form with the full-length sequence *in vivo*, but under the stimulation of pathogen-associated molecular patterns (PAMPs) or damage-associated molecular patterns (DAMPs), Caspase-1/3/7 are activated and cleave GSDME in the linker region to release the NT domain, and then GSDME-NT oligomerization can penetrate cellular membrane, destroy cytomembrane integrity and lead to pyroptosis. Finally, the membrane rupture cells, as well as PAMPs and DAMPs, are phagocytosed by neutrophils to clear pathogenic bacteria and damaged cells (18, 19, 23–26).

Accompanying pyroptosis occurrence, massive cytoplasmic contents such as interleukin-1β (IL-1β), tumor necrosis factor-α (TNF-α), lactate dehydrogenase (LDH) and ATP were released into the extracellular area at the same time (27, 28). As a key pro-inflammatory cytokine, IL-1β can trigger an inflammatory reaction at the site of pathogenic infection to clear pathogens (29–32). However, IL-1β, without signal peptide, could not be directly secreted to the outside of cell. Previous studies have shown that the predicted maximum diameter of IL-1β was 4.5 nm, and the intracellular IL-1β could be released to the extracellular through the pores formed by gasdermin (33, 34). However, LDH could not pass through the gasdermin pore because the predicted maximum diameter of LDH tetramer was 9.6 nm, and it could only be released upon membrane rupture (35). Hence, only IL-1β, not LDH, is released from intracellular area to extracellular area, which can be used as biological indicator of membrane pore formed by GSDME in fish (35). Both IL-1β and LDH are released into the extracellular simultaneously, and it is indicated that cell membrane rupture and pyroptosis appear.

Previous studies have shown that zebrafish *Dr*GSDMEa and *Dr*GSDMEb, turbot (*Scophthalmus maximus*) *Sm*GSDMEb and tongue sole (*Cynoglossus semilaevis*) *Cs*GSDME could be cleaved by caspases and formed the membrane pores to cause pyroptosis (16–19). However, up to now, the function of GSDMEa in common carp is still unclear. Herein, we investigated the roles of *Cc*GSDMEa in the pore-formation of cellular membrane and regulation of IL-1β secretion after *A. hydrophila* infection. This study aims to clarify the roles of *Cc*GSDMEa in immune response of common carp and provide a new insight into protecting fish against pathogen infection.

## Materials and methods

### Experimental animals and cell culture

Experiments involving live animals were conducted in accordance with the ‘‘Regulations for the Administration of Affairs Concerning Experimental Animals’’ promulgated by the State Science and Technology Commission of Henan Province. This study was approved by the Animal Care and Use Ethics Committee of the Henan Normal University.

The healthy common carp (23 ± 2 g, 11 ± 1 cm) were from the aquaculture base in Zhengzhou, Henan province. Before the experiment, the fish were randomly grouped with 50 fish in each group, and then acclimated in 200 L tanks for two weeks. During acclimation, the fish were maintained at 25 ± 2 °C, and pH 7.0 ± 0.2, with the dissolved oxygen concentration of 6.0 ± 0.2 mg/L and natural light/dark cycle, and 1/3 water in tank was exchanged with the fresh aerated water twice a week. The fish were fed with pellets at 5% body weight. The common carp (4% of stock) were randomly sampled for bacterial or virus detection, and no pathogen was detected in the sampled fish, the experimental fish were considered to be healthy (36).

Mycoplasma-free Human Embryonic Kidney (HEK) 293T cells were cultured in DMEM medium (Hyclone, USA) supplemented with 10% (v/v) Fetal bovine serum (FBS, Gibco, USA), 2 mM L-Glutamine, and 4.5 g Glucose, at 37 °C in a 5% CO_2_ incubator. For mycoplasma-free *Epithelioma papulosum cyprini* (EPC) cell, the M199 medium (Hyclone, USA) supplemented with 10% FBS, 1000 mg D-Glucose, 2200 mg NaHCO_3_, and 100 mg L-Glutamine was used, and cultured at 25˚C in an incubator with 5% CO_2_. The cells were passaged every 2-3 d at a split ratio of 1:3 when the cells reached 90% confluency.

### Bacterial strain culture

*A. hydrophila*, which was stored in our laboratory and isolated from common carp (15), cultured on Luria-Bertani broth (LB) plates at 28°C overnight, and single colony of each strain was then inoculated into 5 mL LB medium at 28 °C with constant shaking (180 rpm), and then sub-cultured in 100 mL LB medium with the ratio of 1:100 at 28 °C at 180 rpm until the absorbance values (OD_600_) was 0.6 (37). The half lethal concentration (LC_50_) of bacteria was 7 × 10^7^ CFU/mL. The bacteria were harvested by centrifugation at 8000 rpm at 4 °C for 10 min, washed three times with 0.75% physiological saline (0.75% NaCl), and then resuspended in 0.75% NaCl with the concentration of 7 × 10^6^ CFU/mL.

### RNA extraction and cDNA synthesis

Total RNA was extracted from the samples using RNA extraction reagent RNAiso (TaKaRa, Japan) on the basis of the protocol of manufacture. Total RNA was dissolved in the RNase free water (Sangon, China). The concentration was determined by NanoDrop 2000 spectrophotometer (Thermo company, USA), and the purity was detected through 1% agarose gel electrophoresis. First-strand cDNA used for gene cloning was synthesized using the PrimeScript™ II 1st Strand cDNA Synthesis Kit (TaKaRa, Japan), and the PrimeScript™ RT Master Mix was used to synthesize cDNA template for quantitative Real Time PCR (qRT-PCR) (Vazyme, China). The reverse transcription products were stored at -20°C until used.

### Gene cloning and bioinformatics analysis

The open reading frame (ORF) amplification of *CcGSDMEa* was performed in a 20 μL reaction system, which was made up of 1 μL cDNA template, 1 μL forward primer, 1 μL reverse primer, 10 μL 2 × Taq Master Mix, and 7 μL ddH_2_O. The PCR procedure was set as follows: 5 min at 94 °C for pre-denaturation, 34 cycles (30 s at 94°C for denaturation, 30 s at 58 °C for annealing, and 120 s at 72 °C for extension), and 10 min at 72°C for final extension. The products were analyzed on 1.5% agarose electrophoresis and purified by DNA gel extraction kit (Omega, USA). The purified DNA was ligated into the pMD-19T vector (Takara, Japan), and then transformed into *Escherichia coli* DH5α (Biomed, China). Positive clones were sequenced in Sangon Biotech Company (Shanghai, China).

The rapid amplification of cDNA end (RACE) method was used to amplify the 3’ and 5’ end sequences of *CcGSDMEa*. According to the instruction of PrimeScript™ II 1st Strand cDNA Synthesis Kit, the Oligo dT Primer was replaced by the 3’ RACE Olig(T)-adaptor for reverse transcription to obtain the 3’ RACE template. With regard to 5’ RACE amplification template, the obtained cDNA product was purified with E.Z.N.A. Cycle-pure Kit (Omega, USA), and Poly (A) tail was added at the 3’ end after purification. A total of 50 μL reaction system was as follows: cDNA 10 μL, 5 × TdT buffer 10 μL, 1% BSA 5 μL, dATP (10 mmol/L) 2.5 μL, TdT enzyme 1 μL, and ddH_2_O 21.5 μL. Reaction condition was at 37 °C for 30 min and at 80 °C for 3 min. The obtained final product was 5’ RACE template. The PCR amplification was performed using the primers of 3’/5’ -Out and 3’ RACE Adaptor/5’ RACE Olig(T)-Adaptor for the first round PCR, and then followed by the nested PCR with the primers of 3’/5’ -In and 3’/5’ RACE Adaptor using the first round PCR products (diluted 50 times) as the template. The reaction system and PCR procedure were same as ORF amplification, except only 20 cycles for first round PCR. The primers used for gene cloning were shown in **Table 1**.

**Table 1:** Primers used for gene cloning and qRT-PCR in this study.

Primer names	Sequences (5’-3’)	Usage
*CcGSDMEa* ORF-F	ATGTTTGATAAAGCGACAAAG	ORF amplification
*CcGSDMEa* ORF-R	TCATGCAGCAACAAAGGATGC	
*CcGSDMEa* 5’-Out	CATTGAGTTTGAATCCTGTGGGTC	5’ RACE
*CcGSDMEa* 5’-In	CACCACCGCAAGCACCTG	
*CcGSDMEa* 3’-Out	GGATGCTGGACTGGACCTGC	3’ RACE
*CcGSDMEa* 3’-In	CAATGTTTTGCTACGGAAAGAGAATG	
5’ RACE Olig(T)-Adaptor	GACTCGAGTCGACATCG(T)_17_	Adaptor primer for RACE
5’ RACE Adaptor	GACTCGAGTCGACATCG	
3’ RACE Olig(T)-Adaptor	CTGATCTAGAGGTACCGGATCC(T)_14_	
3’ RACE Adaptor	CTGATCTAGAGGTACCGGATCC	
*CcGSDMEa* qRT-PCR-F	TGCTTTGGTGGATAGACTTGAGA	qRT-PCR
*CcGSDMEa* qRT-PCR-R	GTGGTAAGTCCACTCTTGTCAAG	
*CcIL-1β* qRT-PCR-F	CAGAGCAACAAACTAAGTGACGAG	
*CcIL-1β* qRT-PCR-R	GTGACCCGAATGACAGCCTC	
*18S* qRT-PCR-F	GAGACTCCGGCTTGCTAAAT	
*18S* qRT-PCR-R	CAGACCTGTTATTGCTCCATCT	

The amplified ORF was overlapped with the 3’/5’ terminal sequences to obtain the full length of *CcGSDMEa* cDNA sequence, implemented in software DNAMAN. CLC Main Workbench 6.8 was used to translate the amino acid sequences. Theoretical isoelectric point (*pI*) and molecular weight (MW) were respectively calculated on the basis of deduced amino acid sequences by the Compute *pI*/MW software (https://web.expasy.org/compute_pi/). The domains were predicted and annotated with Simple Modular Architecture Research Tool-SMART (http://smart.embl-heidelberg.de/), and the three-dimensional structure of protein was predicted by I-TASSER (https://zhanggroup.org/I-TASSER). Homologous sequences were searched and analyzed by the BLAST algorithm of NCBI (http://blast.ncbi.nlm.nih.gov/Blast.cgi). Multiple sequence alignments were carried out using the DNAMAN 8.0, and phylogenetic tree was constructed using the neighbor-joining method, implemented in MEGA 7.0 program with a bootstrap test of 1000 replicates.

### Spatial expression analysis by qRT-PCR

Various tissues including gill, liver, spleen, head kidney, trunk kidney, heart, intestine, brain, muscle, and skin were taken aseptically from six healthy fish (23 ± 2 g), respectively. Total RNA extraction and cDNA synthesis were performed as described above. The AceQ qPCR SYBR Green Master Mix (Vazyme, China) and LightCycler^®^ 480 II instrument (Roche, Switzerland) were used for qRT-PCR to detect gene expression at mRNA level. The qRT-PCR was performed in a 20 μL reaction system (10 μL 2 × AceQ qPCR SYBR Green Master Mix, 1 μL specific primers, 3 μL cDNA template and 6 μL ddH_2_O), and cycling program (180 s at 95 °C for pre-incubation, followed by 40 cycles of 95 °C for 10 s and 60 °C for 30 s for amplification). The amplification efficiency of qRT-PCR primers for *CcGSDMEa* and *CcIL-1β* gene was 97.34% and 99.07%, respectively. The *18S* was used as the reference gene, and the amplification efficiency was 100.8% (**Table 1**). The results were analyzed by the 2^-ΔΔCt^ method.

### *A. hydrophila* challenge and sampling

The healthy common carp (23 ± 2 g) were randomly divided into two groups (the treated group and the control group), with 50 fish each group. Fish were injected intraperitoneally with 100 μL *A. hydrophila* suspension at a concentration of 7 × 10^6^ CFU/mL in treated group. In the control group, the fish were injected with 0.75% NaCl in the same volume. The experiments were carried out in triplicate. The fish were not fed during the experiment period and maintained under the same conditions with those during the acclimation period. The mortality of fish was observed and recorded every day. After *A. hydrophila* challenge, six fish from each group were randomly selected at 0 h, 3 h, 6 h, 12 h, 24 h, 48 h and 96 h, respectively, which were anesthetized with ethyl 3-aminobenzoate (MS-222, Sigma, USA), and then sampled. Blood was collected from the caudal vein and centrifuged at 3000 rpm at 4 °C for 10 min to collect serum, which was used for Enzyme-Linked Immunosorbent Assay (ELISA). The tissues (gill, liver, spleen, head kidney, intestine and skin) were sampled immediately and stored in liquid nitrogen. Total RNA extraction, cDNA synthesis, and qRT-PCR were carried out as described above.

### Plasmid construction

The different forms of *Cc*GSDMEa were cloned into His-tagged pET-32a vector or GFP-tagged pEGFP-C1 vector through Hind III and KpnI sites to generate r*Cc*GSDMEa-FL (1-532 aa) or GFP-*Cc*GSDMEa-FL, r*Cc*GSDMEa-NT (1-252 aa) or GFP-*Cc*GSDMEa-NT and r*Cc*GSDMEa-CT (257-532 aa) or GFP-*Cc*GSDMEa-CT recombinant plasmids. *Cc*Caspase-1a/1b/3a/3b/7a/7b sequences were inserted into N-DmrB-pcDNA3.1-3HA expression vector through EcoR I and KpnI sites, respectively. The *Cc*GSDMEa, *Cc*Caspase-1a/1b/3a/3b/7a/7b, pET-32a (+) vector, pEGFP-C1 vector and N-DmrB-pcDNA3.1-3HA vector were digested by restriction enzymes (New England Biolabs, USA), and then they were purified and ligated together by the T4 ligation enzyme (TakaRa, Japan). The recombinant plasmids were transferred into *E. coli* strain DH5α, and the positive clones were verified by sequencing. The primers used to construct plasmids were listed in **Table 2**. To remove the endotoxin, the constructed recombinant eukaryotic plasmids were extracted with an EndoFree Plasmid Kit following the instruction of manufacturer (Omega, USA).

**Table 2:** Primers used for plasmid construction in this study.

Primer names	Sequences (5’-3’)
r*Cc*GSDMEa-F	GGGGTACCATGTTTGATAAAGCGAC (KpnI)
r*Cc*GSDMEa-NT-R	CCCAAGCTTGGGATGGGGATTCTGGGAAAT (Hind III)
r*Cc*GSDMEa-CT-F	GGGGTACCGGTCAGTGGCCACTGATC (KpnI)
r*Cc*GSDMEa-R	CCCAAGCTTTCATGCAGCAACAAAGG (Hind III)
GFP-*Cc*GSDMEa-F	CCCAAGCTTCGATGTTTGATAAAGCGAC (Hind III)
GFP-*Cc*GSDMEa-NT-R	GGGGTACCGAAAGTGGGATGGGGATT (KpnI)
GFP-*Cc*GSDMEa-CT-F	CCCAAGCTTCGGGTCAGTGGCCACTGATC (Hind III)
GFP-*Cc*GSDMEa-R	GGGGTACCTCATGCAGCAACAAAGGATGC (KpnI)
*Cc*Caspase-1a-3HA-F	CGGGTACCATGGCGAAGAGTACTAAGGAG (KpnI)
*Cc*Caspase-1a-3HA-R	GGGAATTCTGAGAGTCCGGGGAACAGGTAG (EcoR I)
*Cc*Caspase-1b-3HA-F	GGGGTACCATGGACATCAAAAGAGTTATG (KpnI)
*Cc*Caspase-1b-3HA-R	CGGAATTCTTACATGAGTCCAGGGAACAGG (EcoR I)
*Cc*Caspase-3a-3HA-F	GGGGTACCATGGATTCGTATCTCACAG (KpnI)
*Cc*Caspase-3a-3HA-R	CGGAATTCTTATTTAGAGAAATAGAG (EcoR I)
*Cc*Caspase-3b-3HA-F	CCAAGCTTATGAACGGAGACTGCGTG (Hind III)
*Cc*Caspase-3b-3HA-R	CGGAATTCTCAAGCAGTGAAGTACATC (EcoR I)
*Cc*Caspase-7a-3HA-F	GGGGTACCATGATGTCTTGTAAATTTC (KpnI)
*Cc*Caspase-7a-3HA-R	CGGAATTCTCAGCTGATGGAGCTCTTC (EcoR I)
*Cc*Caspase-7b-3HA-F	GGGGTACCATGGTTTCGCTTTCTCCG (KpnI)
*Cc*Caspase-7b-3HA-R	CGGAATTCTCAGTTGAAGTAGAGCTC (EcoR I)

The underlined letters indicated restriction enzyme site.

### *Cc*GSDMEa cleavage assay

A total of 5 × 10^6^ EPC cells were seeded per well in six-well plates for 16 h, then co-transfected with GFP-*Cc*GSDMEa-FL (2.5 μg) and *Cc*Caspase-1a/1b/3a/3b/7a/7b (2.5 μg) plasmids using Lipofectamine 3000 Reagent (Thermo Fisher Scientific, USA) according to the instructions when the cells reached 90% confluency. 36 h later, the EPC cells were exposed to 1 μM AP20187 (a chemical inducer of dimerization, which could penetrate the cell membrane and induce the DmrB fusion protein to dimerize. Selleck, China) for 6 h to mediate *Cc*Caspase-1a/1b/3a/3b/7a/7b dimerization (38, 39). Next, the cells were collected, and the total proteins were extracted using the protein extraction kit (Solarbio, China) according to the specification. In detail, the EPC cells were lysed in lysis buffer supplemented with 1% PMSF, protease inhibitor and phosphatase inhibitor on ice for 15 min, and then centrifuged at 12000 rpm at 4 °C for 10 min. Protein concentration was detected by BCA assay kit (Solarbio, China). Subsequently, the protein was mixed with 5 × protein loading buffer (Solarbio, China) and boiled for 10 min, and then used for western blotting.

### Immunocytochemical staining

The HEK293T cells (5 × 10^5^ cells/well) were seeded on coverslips into 6-well cell culture plates overnight, and the plasmids expressing GFP-*Cc*GSDMEa-FL, GFP-*Cc*GSDMEa-NT and GFP-*Cc*GSDMEa-CT (1000 ng/well) were transfected with Lipofectamine 3000 Reagent when the cells reached 70% confluency, and the pEGFP-C1 vector was used as the control.

At 24 h after transfection, the cells were collected, and washed three times with sterile 1 × PBS buffer (Solarbio, China), and then fixed with 4% paraformaldehyde at room temperature for 1 h, washed three times. The cells were incubated with 0.1% Hoechst 33342 (Invitrogen, USA) for ten minutes for the sake of nucleus staining, or incubated with 0.1% DiI (Beyotime, China) for 10 min for the sake of cell plasma membrane staining, and then washed three times. The cell coverslips were mounted on glass slides with Antifade Mounting Medium (Beyotime, China) and air-dried at room temperature. The fluorescence images of cells were observed under a fluorescence microscope (Zeiss, Germany). In addition, the HEK293T cells were fixed with 2.5% glutaraldehyde at 4 °C overnight, and dehydrated using series gradient alcohol and tertiary butanol, and then the integrity of cytomembrane was observed under a biotypic scanning electron microscope (JEOL, Japan).

### Assay for antibacterial effect and cytotoxicity

The different forms of r*Cc*GSDMEa recombinant plasmids were transferred into *E. coli* strain BL21 (DE3), and the transformants were cultured in LB with 100 μg/mL Ampicillin (Amp), shaken at 200 rpm at 37 °C until OD_600_ to 0.6. The bacteria diluted at 10^4^ times, were evenly spread with 50 μL on LB agar plates with or without 0.1 mM IPTG, and then cultured at 37 °C overnight. The CFU on the plates was counted and statistically calculated. At the same time, the growth curve of *E. coli* was tested by measuring the OD_600_ after induction by IPTG.

The cytotoxicity was monitored by evaluating the release of LDH from cells. The cell culture medium was centrifuged at 400 g for 5 min at 4°C, and the supernatant was collected. The LDH content released from the cells was detected according to the instruction of the Lactate Dehydrogenase Cytotoxicity Detection Kit (Beyotime, China). The untransfected cells were used as the blank background control, and the LDH released from the cells treated with LDH release agent was used as the positive control with maximum enzyme activity. Each sample was tested in triplicate. Cytotoxicity (%) = (treated sample A_490_ - blank background control A_490_)/(maximum enzyme activity A_490_ - blank background control A_490_) × 100.

### Assay for bacterial clearance

The healthy fish (9 ± 0.5 g) were randomly divided into five groups (i.e., Control group: PBS; Knockdown groups: siControl group and si*Cc*GSDMEa group; Overexpression groups: pEGFP-C1 group and GFP-*Cc*GSDMEa-FL group). The primers of siRNAs (si*Cc*GSDMEa-F/R, 5’-GGA UCU AUA GAG UUG GGA ATT-3’, 5’-UUC CCA ACU CUA UAG AUC CTT-3’; siControl-F/R, 5’-UUC UCC GAA CGU GUC ACG UTT-3’, 5’-ACG UGA CAC GUU CGG AGA ATT-3’) were designed and synthesized by Sangon Biotech Company (Shanghai, China). For the different knockdown groups, the fish were intramuscularly injected with 100 μL (10 μg) siControl or si*Cc*GSDMEa, respectively. The fish were intramuscularly injected with 100 μL (10 μg) GFP-*Cc*GSDMEa-FL or pEGFP-C1 plasmid in different overexpression groups, respectively (29, 40). In the control group, the fish were injected with 100 μL PBS by the same method. The experiments were carried out in triplicate. After 3 and 7 d, the tissues (gill, liver, spleen, trunk kidney and head kidney) were sampled in the aseptic condition and used for analyzing the efficiency of knockdown or overexpression by RT-qPCR.

In knockdown groups, overexpression groups and the control group, the fish were intraperitoneally injected with 100 μL *A. hydrophila* suspension (7× 10^6^ CFU/mL) after knockdown or overexpression 3 d. The experiments were carried out in triplicate. After challenge for 24 h, the tissues were sampled respectively in the aseptic condition, and used to count the bacterial number in samples by plate count and detect the expression of *CcIL-1β* by RT-qPCR. Blood was collected from the caudal vein and centrifuged at 3000 rpm at 4 °C for 10 min to collect the serum, which was used for ELISA to measure the content of *Cc*IL-1β.

### Assay for *Cc*IL-1β secretion

EPC cells (5 × 10^6^ cells/well) were seeded into 6-well cell culture plates overnight, and then the siControl, si*Cc*GSDMEa, pEGFP-C1 and GFP-*Cc*GSDMEa-FL plasmids (1000 ng/well) were transfected when the cells reached 90% confluency, respectively. The untransfected cells were used as the control. The efficiency of knockdown or overexpression *in vitro* was analyzed after 24 h and 48 h, respectively. The untransfected cells and the cells transfected successfully with the above plasmids were divided into two subgroups, respectively. One group was stimulated by 10 μL *A. hydrophila* (1×10^4^ CFU/well), and the other group was added with isometric PBS. After 3 h, the content of LDH and *Cc*IL-1β in cell culture medium was assessed to determinate the role of *Cc*GSDMEa in regulating IL-1β secretion.

### ELISA

The levels of *Cc*GSDMEa and *Cc*IL-1β in serum were measured according to the method of ELISA (6, 40). Briefly, 96-well Immuno-Maxisorp plates were coated with the serum (1:1) in 2× coating buffer (0.05 M carbonate buffer, pH 9.6) overnight at 4 °C. Next, 10% skimmed milk powder was used as the blocking agent at 37 °C for 2 h, and then were subsequently washed three times (wash buffer: 20 mM PBST, 274 mM NaCl, 5.4 mM KCl, 20 mM Na_2_HPO_4_, 3.6 mM KH_2_PO_4_, 0.5 mL Tween-20, PH 7.4). Polyclonal antibodies against *Cc*GSDMEa or *Cc*IL-1β (1: 500, prepared in our laboratory) were added to the wells to incubation at 37 °C for 2 h and an addition wash. After incubation with horseradish peroxidase labeled Goat anti-Mouse IgG/HRP (Solarbio, China) at 37 °C for 1 h, the plates were washed again. 3,3’,5,5’ -tetramethylbenzidine (TMB, Sigma, USA) was used as the substrate for color development at 37 °C for 30 min. The reaction was terminated by adding 50 μL of termination solution (2M H_2_SO_4_). The OD_450_ absorbance value was detected in a microplate reader (PerkinElmer, USA) within 10 min, and the amount of excretive *Cc*GSDMEa and *Cc*IL-1β (pg/mL) was estimated by comparing to reference curves constructed using the corresponding standards.

### Western blotting analysis

The protein samples (30 μg) were separated by SDS-PAGE before being transferred to a PVDF membrane (Millipore, USA) according to traditional protocol. The membranes were blocked in 5% w/v nonfat dry milk in TBST and then washed three times using TBST for 10 min every time. The mouse anti-GFP Ab (1:10,000, GenScript, China) or mouse anti-β-actin Ab (1:10,000, Beyotime, China) was incubated overnight at 4°C and washed another three times, then followed by incubation with the appropriate secondary goat anti-mouse IgG-HRP Ab (1:5000; Solarbio, China) and washed three times again, whereafter detected by super ECL detection reagent (Millipore, USA) using gel imaging system (Biorad, USA).

### Statistical analysis

All values in this study were presented as means ± standard deviation (M ± SD) (n=3). The data statistical analysis was performed by SPSS 20.0 and GraphPad Prism 5.0. The statistical significance was determined using Student’s *t* test and one-way ANOVA followed by Tukey’s test. Significant difference was set as *P <*0.05 (*), *P <*0.01 (**), or *P <*0.001 (***).

## Result

### Structural analysis of *Cc*GSDMEa

**Figure 1:**
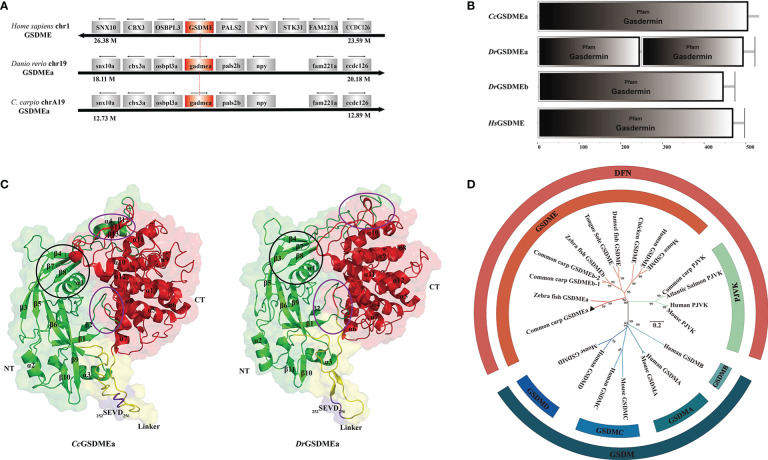
Structural feature and phylogenetic evolution of *Cc*GSDMEa. **(A)** Gene synteny and chromosomal location analysis of *CcGSDMEa*. The genes adjacent to GSDME loci on human chromosome 1, zebrafish chromosome 19, common carp chromosome A19 are shown. Arrows indicate gene orientation. The small arrows above the gene indicate the direction in which the gene is transcribed. **(B)** Structure domains of *Cc*GSDMEa, *Dr*GSDMEa/b, and *Hs*GSDME predicted by SMART. **(C)** The tertiary structures of *Cc*GSDMEa and *Dr*GSDMEa modeled by I-TASSER. The NT domains and CT domains are colored green and red, respectively. The motifs of tetrapeptide _252_SEVD_256_ specifically recognized by caspases are marked in purple, and the linker regions are labeled in yellow. Secondary structure element α-helices and β-strands are indicated in images. The inter-domain interfaces are highlighted by purple ellipses. The α1, β3, β4, β7 and β8 marked by the black circle form the structure of the membrane inserted N-terminal domain. **(D)** Phylogenetic relationships of GSDM family members. The phylogenetic tree was constructed by MEGA (version 7.0) using the neighbor-joining method. The reliability of each node was estimated by bootstrap test with 1000 replications. The accession numbers of selected GSDM protein sequences from the National Center for Biotechnology Information Reference Sequence database (http://www.ncbi.nlm.nih.gov/RefSeq/) are shown in **Table 3**.

In this study, *CcGSDMEa* (GenBank accession number: ON981707), a homologous *GSDME* gene, was obtained from common carp, which is distributed within 7320 bp genomic fragment on chromosome A19 with 9 exons and 8 introns. The location and transcription direction of the adjacent genes to *CcGSDMEa* were consistent with those of *DrGSDMEa* and *HsGSDME*, and it was suggested that genes adjacent to *CcGSDMEa* loci shared an overall conserved chromosome synteny with those of humans and zebrafish (**Figure 1A**). The full length of *CcGSDMEa* cDNA sequence is 2736 bp with a 1599 bp ORF, a 177 bp 5’-untranslated region and a 960 bp 3’-untranslated region (**Figure S1**). The ORF encoded 532 amino acids was predicted with molecular weight of 58.57 kDa and theoretical isoelectric point of 4.92. It displayed high identities (58.9%) and similarities (76.0%) to zebrafish *DrGSDMEa*, but low identities (29.3% and 24.2%) and similarities (51.2% and 45.8%) to zebrafish *DrGSDMEb* and human *HsGSDME*, respectively (**Table 3**).

**Table 3:** Identity and similarity between *Cc*GSDMEa and other species GSDM sequences.

Gene name	Gene bank No.	Identities	Similarities
*Cc*GSDMEa	ON981707		
*Cc*GSDMEb-1	OP046437	31.9%	52.5%
*Cc*GSDMEb-2	OP046436	30.0%	50.7%
*Dr*GSDMEa	XP_005170134.1	58.9%	76.0%
DrGSDMEb	NP_001001947.1	29.3%	51.2%
*Cs*GSDME	XP_016893587.1	25.9%	52.1%
*Ap*GSDME	XP_022071512.1	27.0%	49.4%
*Gd*GSDME	NP_001006361.2	25.3%	44.6%
*Hs*GSDME	NP_004394.1	24.2%	45.8%
*Mm*GSDME	NP_061239.1	21.9%	45.3%
*Cc*PJVK	XP_018961188.2	15.0%	30.9%
*Ss*PJVK	XP_045556351.1	13.0%	27.0%
*Hs*PJVK	AAI46939.1	24.2%	29.9%
*Mm*PJVK	NP_001074180.1	21.9%	30.4%
*Hs*GSDMD	AAH69000.1	15.4%	32.2%
*Mm*GSDMD	AAH29813.1	13.4%	32.1%
*Hs*GSDMC	AAH35321.1	12.1%	29.8%
MmGSDMC	NP_113555.1	14.0%	32.1%
HsGSDMB	KAI4049241.1	11.7%	30.1%
*Hs*GSDMA	AAI09198.1	14.3%	31.7%
*Mm*GSDMA	NP_067322.1	14.7%	32.3%

To better understand the function of *Cc*GSDMEa, the features of domain and tertiary architecture were characterized. The *Cc*GSDMEa contained one gasdermin domain, which is consistent with the other GSDM family members except for *Dr*GSDMEa (**Figure 1B**). In addition, the *Cc*GSDMEa shared highly conserved tertiary architectures with zebrafish *Dr*GSDMEa, which had a conserved N-terminal pore-forming domain, C-terminal auto-inhibitory domain and a flexible and pliable hinge region (**Figure 1C**). In linker regions, the predicted tetrapeptide motif, which was specifically recognized by caspases, was _252_SEVD_256_. This was the same as the cleavage site of *Dr*GSDMEa. Phylogenetic tree revealed that *Cc*GSDMEa was grouped with zebrafish *Dr*GSDMEa with a high score of bootstrap tests. Interestingly, *Cc*GSDMEa was not grouped with GSDMEb of common carp, but was clustered into a separate clade (**Figure 1D**), which provided us a better clue for future analysis of GSDME evolutionary function in teleost. In addition, both members of the deafness-associated gene (i.e., GSDME and PJVK) were clustered together, and they were distant from the other members (GSDMA, GSDMB, GSDMC and GSDMD).

### Expression profile of *Cc*GSDMEa after *A. hydrophila* challenge

**Figure 2:**
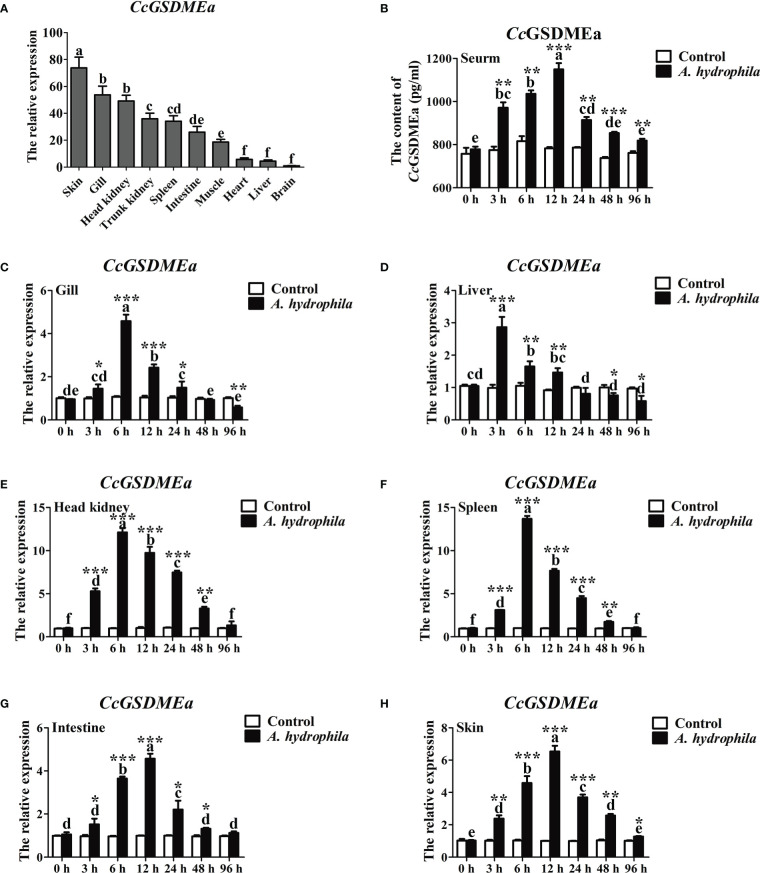
Expression profile of *Cc*GSDMEa after *A. hydrophila* challenge. **(A)** Tissues distribution of *CcGSDMEa* in healthy *C. carpio*. The lowest expression level in brain was chosen as calibration (set as 1), and the relative expression of *CcGSDMEa* in other tissues was represented as fold-changes to the calibration. **(B)** The protein expression levels of *Cc*GSDMEa in serum after *A. hydrophila* challenge. **(C-H)** The mRNA expression levels of *CcGSDMEa* in different tissues after *A. hydrophila* challenge. The control group was chosen as calibration (set as 1). The data were expressed as mean ± SD (n = 3). Significant difference between the different time points were analyzed using one-way ANOVA followed by Tukey’s test, and presented with the different lowercase letters in the group challenged by *A. hydrophila* (*P<*0.05), and the same letter was indicated no significant difference (*P >* 0.05). The significant differences between the *A. hydrophila* challenge group and the control group at the same time point were analyzed by student’s *t* test and indicated with asterisks (*, *P <* 0.05; **, *P <* 0.01, ***, *P <* 0.01).

*CcGSDMEa* was widely expressed in the tested tissues and exhibited a tissue-specific spatial expression pattern. *CcGSDMEa* was highly expressed in the skin and gill, followed by the head kidney and trunk kidney, and the lowest expression was in brain (**Figure 2A**). Moreover, the expression levels in immune-related tissues (head kidney, spleen, skin, intestine and gill) were markedly higher than those in heart, liver and brain.

To further analyze the expression profile of *Cc*GSDMEa in response to bacterial infection, *A. hydrophila* was chosen to stimulate the juvenile common carp. The peaks of *CcGSDMEa* expression were at 3 h in liver, at 6 h in gill, head kidney and spleen, and at 12 h in intestine and skin (**Figures 2C-H**). Although the peaks of mRNA expression levels were different in various tissues and appeared at the different time points, the expression levels of *CcGSDMEa* were increased after *A. hydrophila* infection, compared with the control. The protein expression level of *Cc*GSDMEa showed a trend of increasing at first and then decreasing with the time prolongation in serum, and the expression peak was observed at 12 h (**Figure 2B**). The expression levels among different time points showed significant differences. Meanwhile, the protein expression levels of *Cc*GSDMEa were notably increased at the different time points compared with the control.

### *Cc*GSDMEa-NT was produced from *Cc*GSDMEa cleavage by *Cc*Caspase-1/3/7

**Figure 3:**
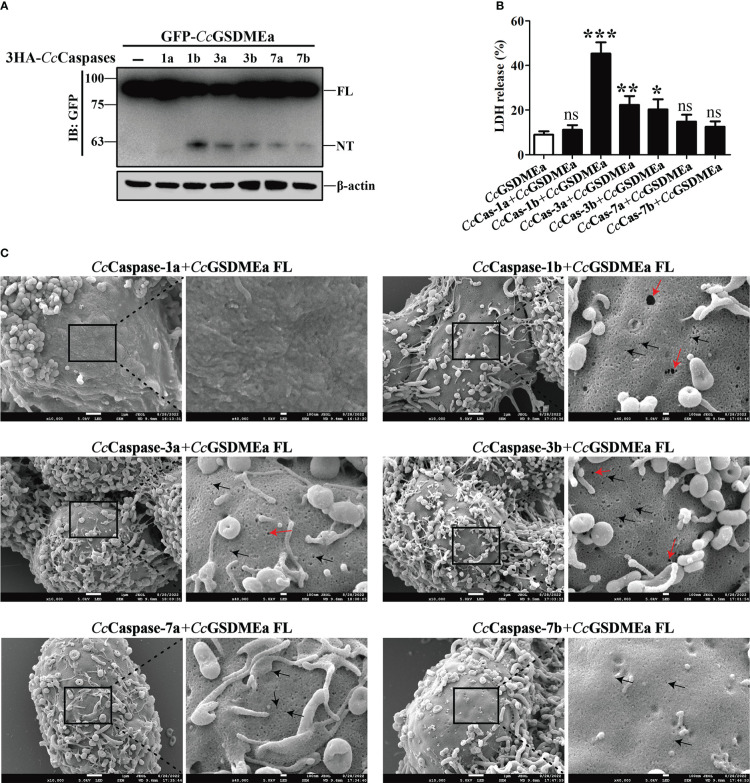
*Cc*GSDMEa-NT was produced from *Cc*GSDMEa cleavage by *Cc*Caspase-1/3/7. **(A)** The western bolting result that *Cc*GSDMEa was cleaved by *Cc*Caspase-1b/3a/3b/7a/7b. **(B)** LDH release from the EPC cells co-transfected with *Cc*GSDMEa and the *Cc*Caspase-1a/1b/3a/3b/7a/7b plasmids. **(C)** Morphological observation of HEK293T cells co-transfected with *Cc*GSDMEa and the *Cc*Caspase-1a/1b/3a/3b/7a/7b plasmids. Red arrows display cell plasma membrane rupture, and dark arrows indicate the gasdermin membrane pores. Values are shown as mean ± SD (n = 3). The LDH release from the cells only transfected with *Cc*GSDMEa was set as control. The significance difference of LDH release between the control cells and the co-transfected cells was analyzed by student’s *t* test and shown as: ns, *P >* 0.05; *, *P <* 0.05; **, *P <* 0.01; ***, *P <* 0.001.

To better understand the cleavage mechanism of *Cc*GSDMEa, each of the *Cc*Caspase-1a, *Cc*Caspase-1b, *Cc*Caspase-3a, *Cc*Caspase-3b, *Cc*Caspase-7a and *Cc*Caspase-7b plasmids was co-transfected with *Cc*GSDMEa-FL into EPC cells, and then treated with the dimerizing drug AP20187 for 6 h. The results showed that *Cc*Caspase-1b, *Cc*Caspase-3a, *Cc*Caspase-3b, *Cc*Caspase-7a and *Cc*Caspase-7b could cleave *Cc*GSDMEa to produce *Cc*GSDMEa-NT, except for *Cc*Caspase-1a (**Figure 3A**). The LDH released from cells co-transfected *Cc*Caspase-1b/3a/3b and *Cc*GSDMEa, were dramatically higher than that released from cells only transfected *Cc*GSDMEa (**Figure 3B**). HEK293T cells were co-transfected with *Cc*Caspase-1a/1b/3a/3b/7a/7b and *Cc*GSDMEa, and the morphology of cell membrane was observed. The results showed that, except for the co-transfection of *Cc*Caspase-1a and *Cc*GSDMEa, membrane pores could be observed in the co-transfected cells (**Figure 3C**). It was indicated that any one of *Cc*Caspase-1b/3a/3b/7a/7b could cleave *Cc*GSDMEa to generate *Cc*GSDMEa-NT, which could oligomerize on the cell membrane and penetrate the plasma membrane.

### The membrane pore formation induced by *Cc*GSDMEa-NT

**Figure 4:**
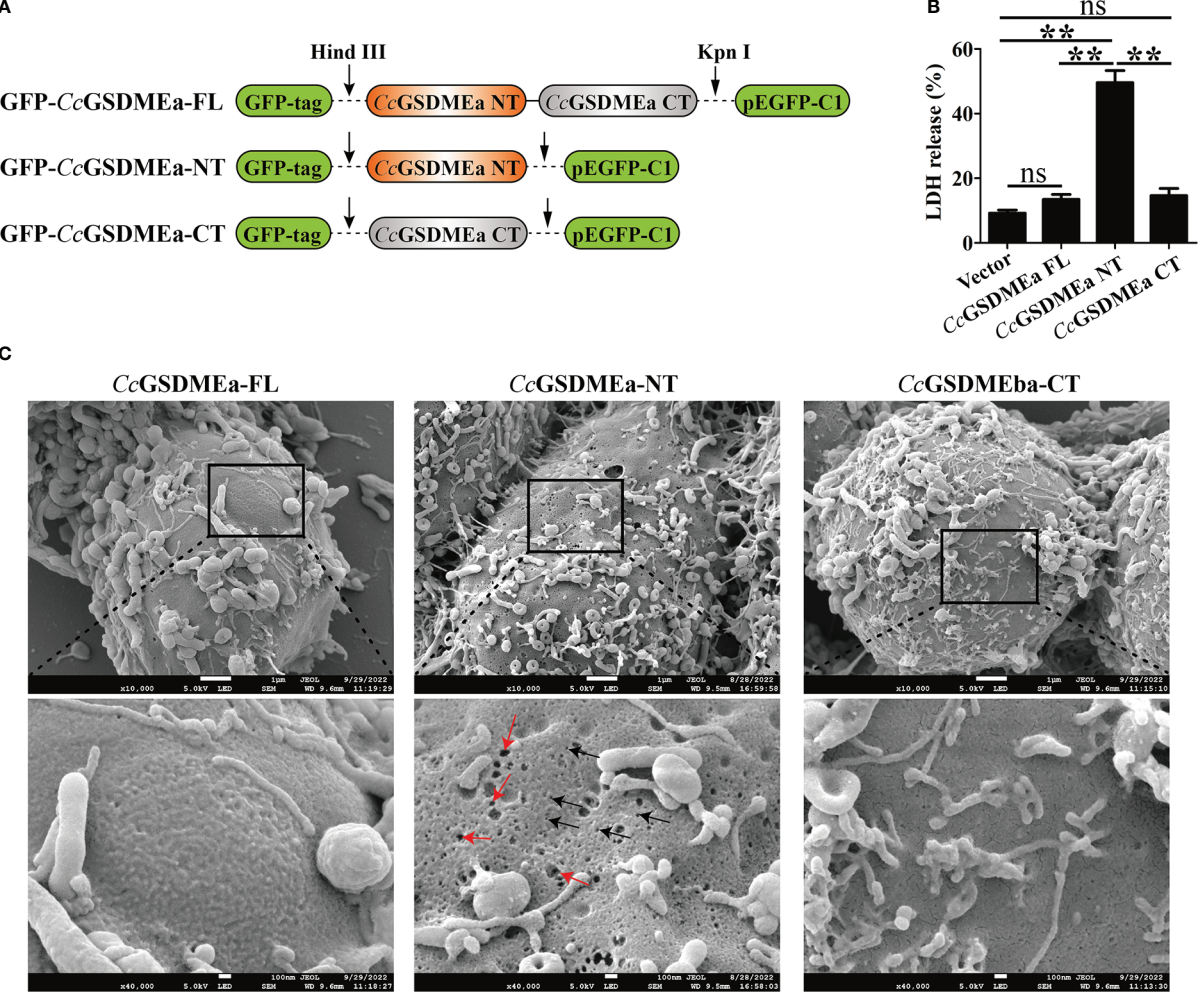
Membrane pore formation induced by *Cc*GSDMEa-NT in HEK293T cells. **(A)** Schematic diagram of the recombinant plasmids of *Cc*GSDMEa-FL (1-532 AA), *Cc*GSDMEa-NT (1-250 AA), *Cc*GSDMEa-CT (257-532 AA) and pEGFP-C1. **(B)** LDH release from cells transfected with 1000 ng of different forms of *Cc*GSDMEa recombinant plasmids. **(C)** Morphological observation of HEK293T cells transfected with different forms of *Cc*GSDMEa recombinant plasmids. Red arrows display cell plasma membrane rupture, and dark arrows indicate the membrane pores induced by *Cc*GSDMEa-NT. The LDH release values are shown as M ± SD (n = 3). The significant differences were analyzed by student’s *t* test, “**” means *P* < 0.01, “ns” means no significant difference.

To explore the pore forming function of *Cc*GSDMEa, the *Cc*GSDMEa-FL/NT/CT recombinant plasmids with GFP-tag were constructed (**Figure 4A**). The different recombinant plasmids of *Cc*GSDMEa were transfected into HEK293T cells to observe the morphological changes and LDH release. The results showed that the release of LDH from cells expressing *Cc*GSDMEa-NT was markedly higher than that of cells expressing *Cc*GSDMEa-FL or *Cc*GSDMEa-CT and pEGFP-C1 vector (**Figure 4B**). Cytomembrane pores and rupture phenomenon were also observed only in the transfected cells with *Cc*GSDMEa-NT plasmids (**Figure 4C**). It was suggested that only *Cc*GSDMEa could punch holes in the membrane and disrupt the integrity of cytomembrane through its NT domain.

**Figure 5:**
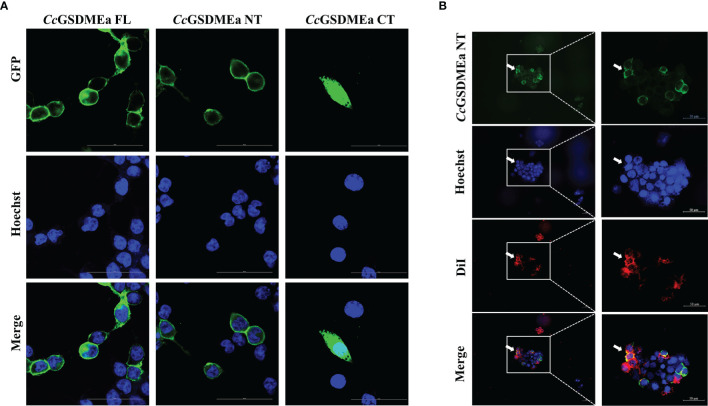
Subcellular localizations of *Cc*GSDMEa different forms. **(A)** Subcellular localizations of *Cc*GSDMEa-FL/NT/CT and Hoechst-stained nucleus in HEK293T cells. **(B)** Subcellular localization of *Cc*GSDMEa-NT, Hoechst-stained nucleus and DiI-stained cytomembrane in HEK293T cells (white arrowheads). The original microscope images were on the left and the locally enlarged images were on the right in **(B)**.

In addition, the subcellular localizations of *Cc*GSDMEa-FL, NT and CT in HEK293T cells were varied (**Figure 5A**). The *Cc*GSDMEa-FL was localized in the cytoplasm and cytomembrane, the *Cc*GSDMEa-NT was only situated in the cytomembrane, and the *Cc*GSDMEa-CT was distributed in the whole cells. We also found that *Cc*GSDMEa-NT and DiI-labeled cytomembrane were completely colocalized (**Figure 5B**), and it was indicated that *Cc*GSDMEa-NT could accumulate on the cellular membrane.

### Bactericidal activity of *Cc*GSDMEa to *E. coli*

**Figure 6:**
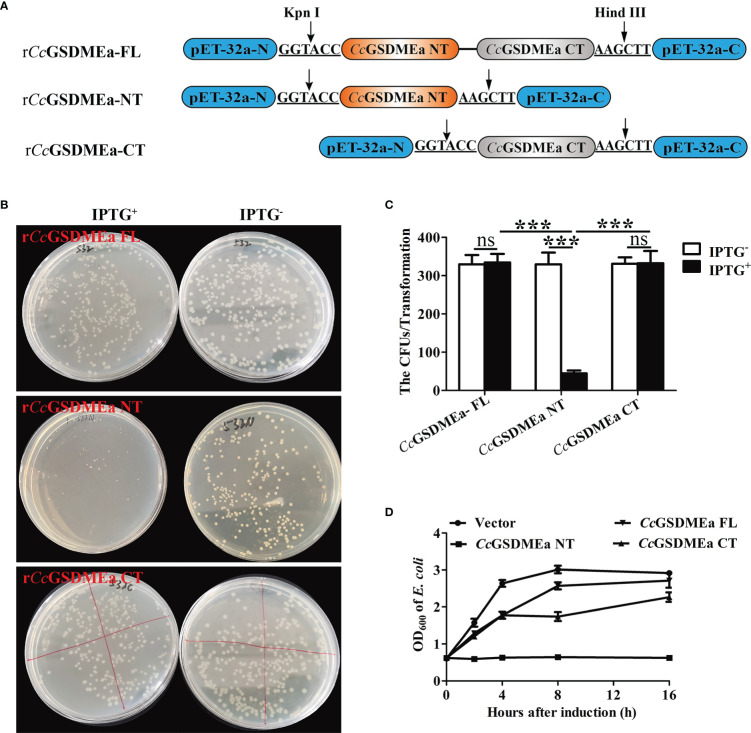
Bactericidal activity of *Cc*GSDMEa to *E. coli*. **(A)** Schematic diagram of the recombinant plasmids of r*Cc*GSDMEa-FL, r*Cc*GSDMEa-NT and r*Cc*GSDMEa-CT. **(B)** Colony growth of *E. coli* expressing r*Cc*GSDMEa-FL/NT/CT. **(C)** Calculation of CFUs on the plates. **(D)** Growth curves of *E. coli* expressing r*Cc*GSDMEa-FL/NT/CT after IPTG induction. Values are expressed as M ± SD (n = 3). The statistical difference was analyzed by student’s *t* test (***, *P* < 0.001; ns, no significant difference).

In order to detect the bactericidal activity of *Cc*GSDMEa, *Cc*GSDMEa-FL, *Cc*GSDMEa-NT and *Cc*GSDMEa-CT were subcloned into pET-32a vector, respectively (**Figure 6A**). The recombinant plasmids r*Cc*GSDMEa-FL/NT/CT were expressed in *E. coli* by IPTG-induced manner. Of note, the number of *E. coli* expressing r*Cc*GSDMEa-NT was markedly reduced compared with that of expressing r*Cc*GSDMEa-FL or r*Cc*GSDMEa-CT after IPTG induction (**Figures 6B, C**). The growth curves of *E. coli* expressing different recombinant plasmids of *Cc*GSDMEa were also been analyzed. The OD_600_ of *E. coli* expressing r*Cc*GSDMEa-NT indicated little change at different time points after IPTG induction, but the OD_600_ values of *E. coli* expressing r*Cc*GSDMEa-FL, r*Cc*GSDMEa-CT and pET-32a vector showed an increasing trend with time prolongation after IPTG induction (**Figure 6D**). It was demonstrated that *Cc*GSDMEa-NT could perform bactericidal function.

### *Cc*GSDMEa promotes the expression of *Cc*IL-1β *in vivo*

**Figure 7:**
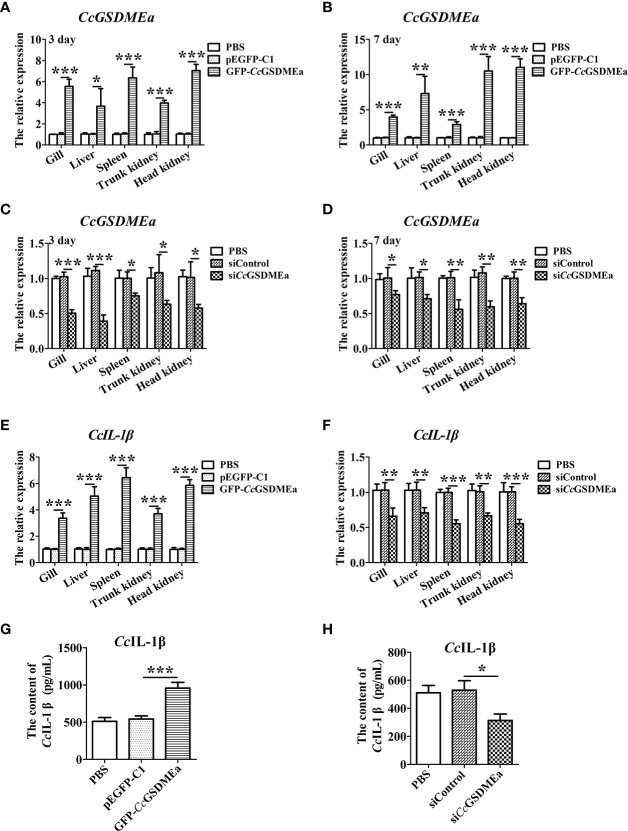
*Cc*GSDMEa promotes the expression of *Cc*IL-1β *in vivo*. **(A-D)** Overexpression efficiency and knockdown efficiency of *Cc*GSDMEa in gill, liver, spleen, trunk kidney and head kidney of *C. carpio*. **(E, F)** The mRNA expression levels of *Cc*IL-1β in the different groups after *A. hydrophila* challenge. **(G, H)** The protein expression levels of *Cc*IL-1β in the different groups after *A. hydrophila* challenge. In each case, the mRNA expression level in the PBS group was chosen as calibration (set as 1). The statistical difference was analyzed by student’s *t* test (*, *P <* 0.05; **, *P <* 0.01; ***, *P <* 0.001).

To better understand the role of *Cc*GSDMEa in regulating *Cc*IL-1β, the *Cc*GSDMEa in fish was knocked down or overexpressed, and the expression of *Cc*IL-1β at the mRNA and protein levels were detected. The knockdown and overexpression of *Cc*GSDMEa were confirmed based on the results of *CcGSDMEa* mRNA expression levels *in vivo*. **Figure 7** showed that the *CcGSDMEa* mRNA expression levels in GFP-*Cc*GSDMEa group were markedly increased compared with the levels in pEGFP-C1 group at 3 d and 7 d (**Figures 7A, B**). However, the expression levels of *CcGSDMEa* in si*Cc*GSDMEa group were lower than those in siControl group (**Figures 7C, D**).

In *Cc*GSDMEa overexpression group, the *Cc*IL-1β expressions at the mRNA and protein levels were notably higher compared with the expression levels in pEGFP-C1 group (**Figures 7E, G**). In *Cc*GSDMEa knockdown group, the expression levels of *Cc*IL-1β decreased compared with the siControl group (**Figures 7F, H**). These results suggested that *Cc*GSDMEa could promote *Cc*IL-1β expression.

### *Cc*GSDMEa prevents *A. hydrophila* colonization *in vivo*

**Figure 8:**
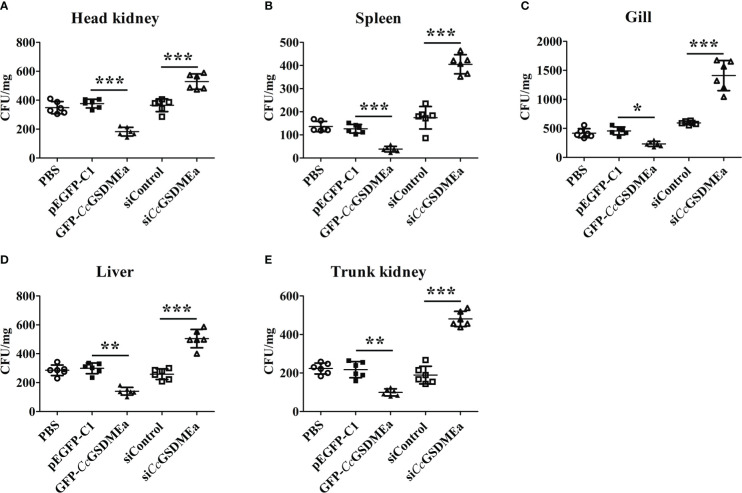
*Cc*GSDMEa prevents *A. hydrophila* colonization *in vivo*. Bacterial burdens in head kidney **(A)**, spleen **(B)**, gill **(C)**, liver **(D)** and trunk kidney **(E)** of different groups infected with *A. hydrophila*. The data are expressed as mean ± SD (n = 3) and analyzed statistically by student’s *t* test (*, *P <* 0.05; **, *P <* 0.01; ***, *P <* 0.001).

As mentioned above, the expression of *Cc*GSDMEa increased in fish when stimulated by *A. hydrophila* (**Figure 2**). After *Cc*GSDMEa was overexpressed or knocked down, the bacterial burdens were examined in tissues of fish after *A. hydrophila* challenge for 24 h. As shown in **Figure 8**, the bacterial burdens were obviously lower in head kidney, spleen, gill, liver and trunk kidney in GFP-*Cc*GSDMEa overexpression group than those in the pEGFP-C1 group. While, in si*Cc*GSDMEa group, the bacterial burdens were observably increased in head kidney, gill, liver, spleen and trunk kidney, compared with those in siControl group. It was indicated that the *Cc*GSDMEa could prevent the colonization of *A. hydrophila* and minish the bacterial burdens in fish.

### *Cc*GSDMEa promotes the secretion of *Cc*IL-1β in EPC cells

**Figure 9:**
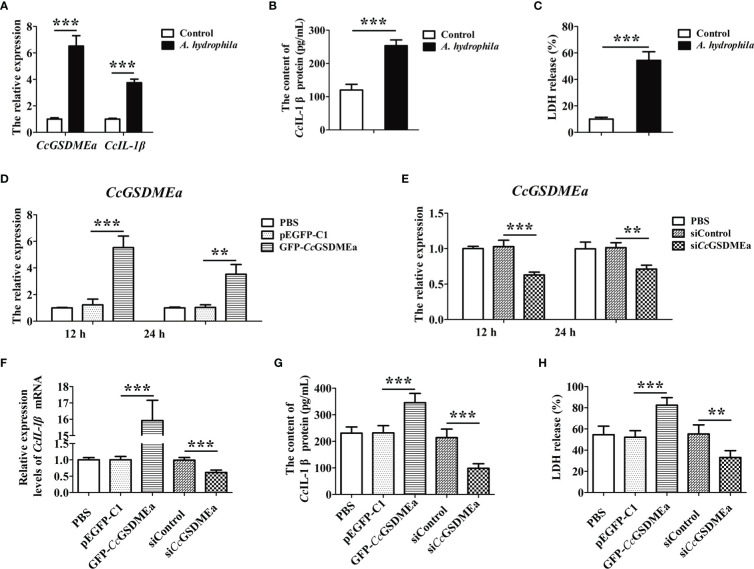
*Cc*GSDMEa promotes the secretion of *Cc*IL-1β *in vitro*. **(A)** The mRNA expression levels of *CcGSDMEa* and *CcIL-1β* in EPC cells after *A. hydrophila* challenge. **(B)** The content of *Cc*IL-1β protein in the cell culture medium after *A. hydrophila* challenge. **(C)** The LDH release in EPC cells after *A. hydrophila* challenge. **(D, E)** The overexpression and knockdown efficiency of *Cc*GSDMEa in EPC cells at 12 h and 24 h. **(F-H)** The mRNA expression levels of *CcIL-1β*, protein secretion contents of *Cc*IL-1β and the LDH release from cells in overexpression or knockdown group after *A. hydrophila* challenge. The data were expressed as mean ± SD (n = 3). The significant differences were analyzed using student’s *t* test (**, *P <* 0.01; ***, *P <* 0.001).

After EPC cells challenged with *A. hydrophila*, the expression levels of *CcGSDMEa* and *CcIL-1β* significantly increased, compared with the control group (**Figure 9A**), it was suggested that *A. hydrophila* could trigger the innate immune responses of EPC cells. Similarly, the contents of *Cc*IL-1β protein in the cell culture medium were also increased after *A. hydrophila* challenge compared with the control group (**Figure 9B**), and it was manifested that *A. hydrophila* challenge could promote the secretion of *Cc*IL-1β. Moreover, the LDH released from EPC cells treated by *A. hydrophila* were observably higher than that in the control group (**Figure 9C**), and indicted that *A. hydrophila* challenge might destroy cytomembrane integrity of EPC cells.

To clarify the effect of *Cc*GSDMEa on *Cc*IL-1β secretion, the *Cc*GSDMEa was overexpressed or knocked down in EPC cells, and then the contents of *Cc*IL-1β protein in cell culture medium were examined after *A. hydrophila* stimulation. As shown in **Figures 9D, E**, the overexpression and knockdown of *Cc*GSDMEa were both affirmed. After *A. hydrophila* challenge, in the *Cc*GSDMEa overexpression group, expression level and secretion content of *Cc*IL-1β and the release of LDH were significantly increased, compared with the pEGFP-C1 group (**Figures 9F-H**). It was illustrated that the overexpression of *Cc*GSDMEa could intensify the effect of *A. hydrophila* challenge on *Cc*IL-1β secretion and LDH release. However, in si*Cc*GSDMEa group, after *A. hydrophila* challenge, the expression level and secretion content of *Cc*IL-1β and the release of LDH were notably decreased compared with the siControl group. It was implied that the knockdown of *Cc*GSDMEa could retard the effect of *A. hydrophila* on *Cc*IL-1β secretion and LDH release. Taken together, these results revealed that *Cc*GSDMEa played an important role in regulating the secretion of *Cc*IL-1β.

## Discussion

The different types of cell death, such as apoptosis, autophagy, necrosis or pyroptosis, mainly depend on the inducing factors and the different stages of the cell cycle. Among these types of programmed cell death, pyroptosis is a newly defined inflammatory programmed cell death that plays an important role in fighting bacterial infections and inflammatory diseases (41). Previous studies have shown that the gasdermin family members were the executors of pyroptosis and played key roles in the innate immunity (18, 42, 43). However, in teleost, only two members of gasdermin family (GSDME and PJVK) have been identified to date, and they were considered as the ancient members of the GSDM family (19, 21, 44). Since PJVK does not perform immune-related functions (35, 45), GSDME is considered to be the only executor of pyroptosis in teleost, and its role of innate immune to resist pathogen invasion has become a research hotspot.

In this study, the *CcGSDMEa* identified from common carp shares conserved chromosomal colinearity and tertiary structure with GSDME in other fish and human (17–19, 24). Similar to the structure of mouse GSDMA3, α1, α4 helix and β1-β2 hairpin in NT domain of *Cc*GSDMEa provide the main surface for binding to the CT domain and then form the major autoinhibitory interactions (44). In addition, α1, β3, β4, β7 and β8 can form the N-terminal structure that inserts into cytomembrane. The α1 is the first α-helix that specifically interacts with phospholipids, and β3, β4, β7 and β8 are four parallel β-strands, which can insert into the membrane. These structural properties of *Cc*GSDMEa might provide the basis for its functional role.

*CcGSDMEa* was ubiquitously expressed in all tested tissues, although the expression levels are different in the varied tissues. In zebrafish, *DrGSDMEa* was highly expressed in intestine, gill, head kidney and spleen, but lowly expressed in brain, heart and muscle (19). The same situation was found in common carp. The high expression of *CcGSDMEa* in immune-related tissues indicated that it played an important role in the immune surveillance system. Studying the expression changes of *Cc*GSDMEa after *A. hydrophila* challenge is helpful to understand the immune response of fish to pathogen infection and provide new insights into the formulation of effective measures to prevent the occurrence of disease. The increased expression levels of *Cc*GSDMEa (mRNA and protein) suggested that *Cc*GSDMEa played an important role in immune response against the invasion of *A. hydrophila*. The peaks of *Cc*GSDMEa expression levels appeared at the early stage of infection, indicated that fish immune response was more active and stronger at the early stage of pathogen infection, and it suggested that the early stage of infection was the best period to clear pathogens in fish.

GSDME could be cleaved by caspases to cause pyroptosis in teleost. For instance, *Dr*GSDMEa could be cleaved by inflammatory caspases (caspase-B and caspase-19b) and apoptotic caspases (caspase-3a/b, caspase-7 and caspase-8a) in zebrafish (16), the *Sm*GSDME could be cleaved by *Sm*caspase in turbot, and *Cs*GSDME could be cleaved by caspase-1/3/7 in tongue sole (18). Similarly, *Cc*GSDMEa was cleaved by inflammatory caspases (*Cc*Caspase-1b) and apoptotic caspases (caspase-3a/b and caspase-7a/b) to release its N-terminal domain to form the pores on cell membrane. Moreover, *Cc*GSDMEa cleaved by *Cc*Caspase-1b/3a/3b also could disrupt the cell membrane. The different LDH release and the different degree of cell membrane destruction of the cells might be due to the different cleavage efficiency of *Cc*GSDMEa by *Cc*Caspase-1b/3a/3b/7a/7b. The tongue sole *Cs*GSDME was cleaved with high efficiency by inflammatory Caspase-1, and with comparatively low efficiency by apoptotic Caspase-3/7 (18). To be different from this result, zebrafish *Dr*GSDMEa was cleaved with high efficiency by Caspase-1/3b and with low efficiency by Caspase-3a/7 (17, 19). This suggested that the efficiency of GSDME being cleaved in fish was not only related to the different of Caspases, but also related to the specificity of species.

Studies have shown that the NT-domain of GSDME could punch holes in the cell membrane and induced pyroptotic bubble formation and membrane lysis (18, 19, 28, 33). The membrane pore formation, cytomembrane rupture and LDH release from cells were observed only in transfected cells with *Cc*GSDMEa-NT plasmid, which was consistent with that found in zebrafish *Dr*GSDMEa and turbot *Sm*GSDMEb (16, 17). In addition, the number of *E. coli* expressing r*Cc*GSDMEa-NT was markedly reduced after IPTG induction, this was consistent with the findings in tongue sole, turbot and zebrafish (16–18). Accumulating evidence suggested that *Cc*GSDMEa-NT could not only punch holes in the membrane of HEK293T cells, but also directly destroy the integrity of bacterial membrane of *E. coli*, and showed that the *Cc*GSDMEa exerted its bactericidal activity and cytotoxicity through its NT domain.

The antibacterial infection mechanism of GSDME remains to be poorly understood in fish at present. In this research, the increased bacterial burdens in various tissues in *Cc*GSDMEa-knockdown group demonstrated that the ability to eliminate the invading bacteria was reduced in host. With respect to *Cc*GSDMEa-overexpression group, it was shown that bacterial loads were obviously decreased in different tissues. These results mentioned above provided the direct evidence that *Cc*GSDMEa played a key role in regulating bacterial clearance in fish. As a key node in the immune and inflammatory networks, IL-1β performs antibacterial defenses through involving in inflammation reaction in innate immunity (29, 30, 32, 46). Our results showed that *Cc*GSDMEa could promote the expression and secretion of *Cc*IL-1β, involve in inflammation reaction, and then help the host eliminate invading pathogens.

**Figure 10:**
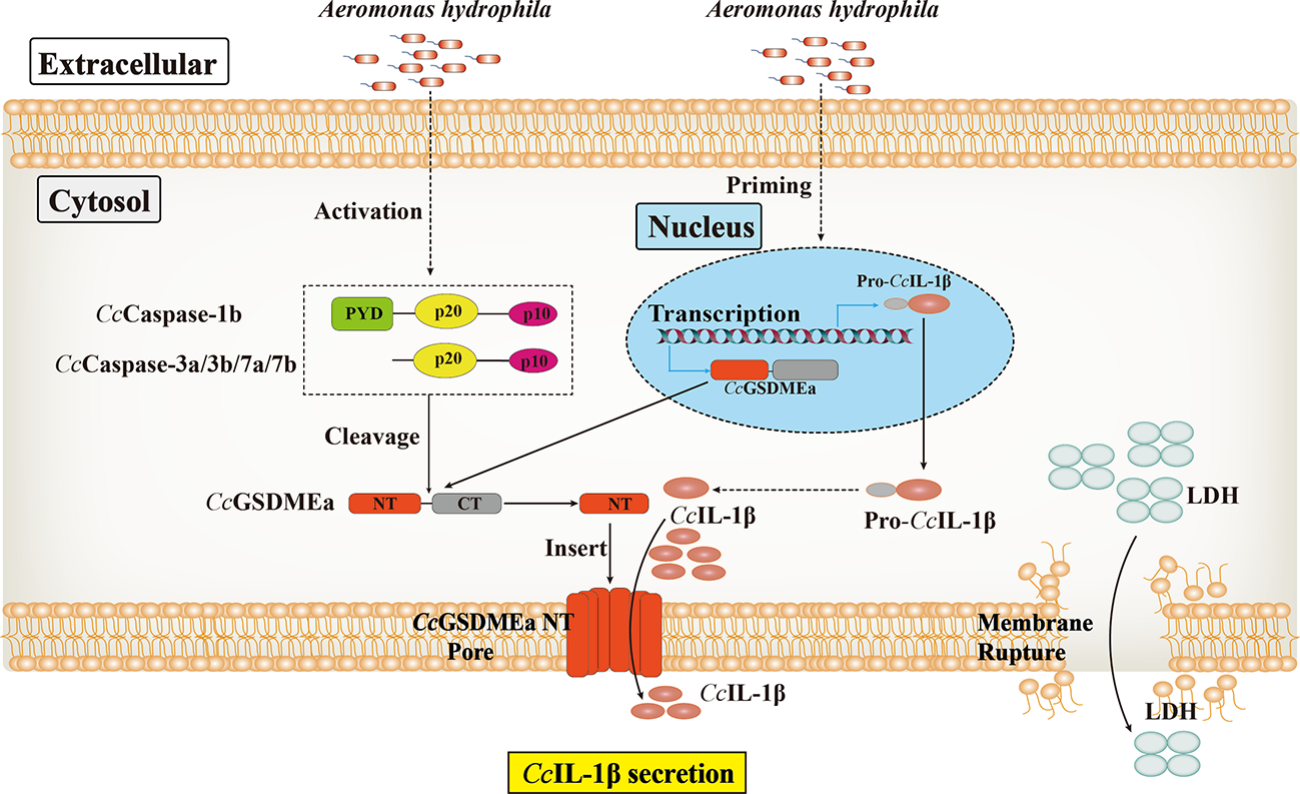
The proposed mode of the regulation role of *Cc*GSDMEa on *Cc*IL-1β secretion in common carp.

To sum up, *Cc*GSDMEa could be cleaved by *Cc*Caspase-1b/3a/3b/7a/7b to generate *Cc*GSDMEa-NT domain, which could oligomerize and translocate to the plasma membrane, where it could form pores to promote pyroptosis. *Cc*GSDMEa was involved in innate immune defense against *A. hydrophila* infection in common carp, through regulating the secretion of *Cc*IL-1β, and it was indicated that *Cc*GSDMEa played an important role in combating bacterial invasion (**Figure 10**). These results will provide a reference for us to further study the immune mechanism to defense against bacterial infection in fish, and also provide new strategies to prevent and control fish diseases for healthy and continuous aquaculture.

## Data availability statement

The datasets presented in this study can be found in online repositories. The names of the repository/repositories and accession number(s) can be found in the article/**Supplementary Material**.

## Ethics statement

The animal study was reviewed and approved by Animal Care and Use Ethics Committee of the Henan Normal University.

## Author contributions

YZ and JZ: conceived, designed and performed the experiments, and analyzed data, as well as wrote the draft manuscript and submitted the manuscript. DQ, FG and YG: carried out the experiments and analyzed data. XJ and LZ: performed data interpretation and acquired finding. XK: designed this study and provided a critical editing of the manuscript and was responsible for forming the finalizing manuscript. All authors contributed to the article and approved the submitted version.
