# Supplementary material for: CcGSDMEa functions the pore-formation in cytomembrane and the regulation on the secretion of IL-lβ in common carp (Cyprinus carpio haematopterus)
Source: Front Immunol. 2023 Jan 6;13:1110322. doi: 10.3389/fimmu.2022.1110322 (PMC9852915; doi:10.3389/fimmu.2022.1110322)

**Supplementary Figure S1 Full-length cDNA sequence and deduced amino acid sequence of *Cc*GSDMEa.** The deduced amino acid sequence is shown under the nucleotide sequence. The 5'-UTR and 3'-UTR are shown in lowercase. The α-helices are wavy underline and β-strands are underlined. The linker domain is highlighted with double underlined, and the Caspase cleavage tetrapeptide motif SEVD is boxed. The numbers of amino acid residues are labeled on the left of the sequence.


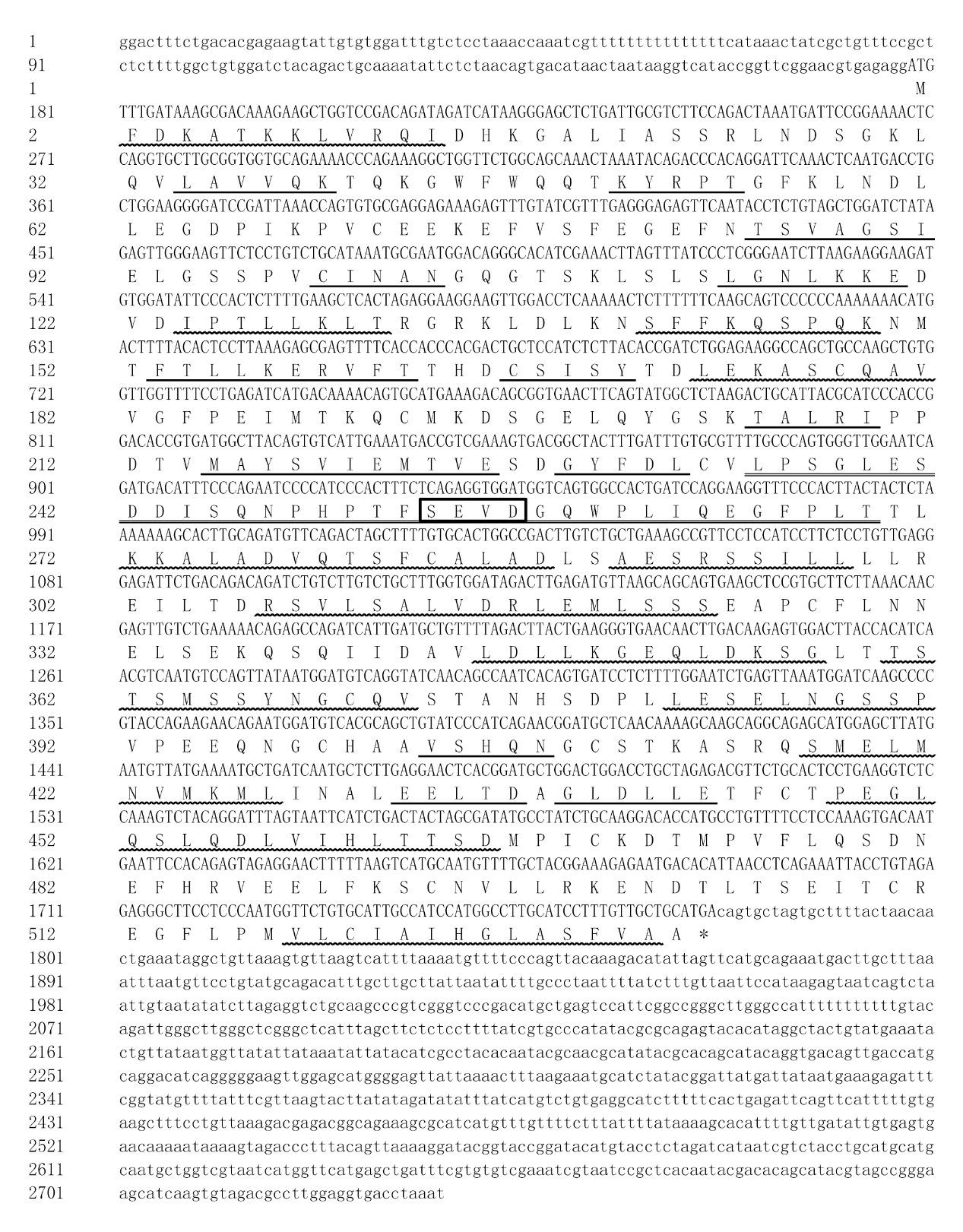

Supplement: Supplementary file 1 [file DataSheet_1.docx]
