# Supplementary material for: CcGSDMEa functions the pore-formation in cytomembrane and the regulation on the secretion of IL-lβ in common carp (Cyprinus carpio haematopterus)
Source: Front Immunol. 2023 Jan 6;13:1110322. doi: 10.3389/fimmu.2022.1110322 (PMC9852915; doi:10.3389/fimmu.2022.1110322)
Supplement: Supplementary file 2 [file Table_1.docx]

> GSDMEa [*Cyprinus carpio*]

GGACTTTCTGACACGAGAAGTATTGTGTGGATTTGTCTCCTAAACCAAATCGTTTTTTTTTTTTTTTCATAAACTATCGCTGTTTCCGCTCTCTTTTGGCTGTGGATCTACAGACTGCAAAATATTCTCTAACAGTGACATAACTAATAAGGTCATACCGGTTCGGAACGTGAGAGGATGTTTGATAAAGCGACAAAGAAGCTGGTCCGACAGATAGATCATAAGGGAGCTCTGATTGCGTCTTCCAGACTAAATGATTCCGGAAAACTCCAGGTGCTTGCGGTGGTGCAGAAAACCCAGAAAGGCTGGTTCTGGCAGCAAACTAAATACAGACCCACAGGATTCAAACTCAATGACCTGCTGGAAGGGGATCCGATTAAACCAGTGTGCGAGGAGAAAGAGTTTGTATCGTTTGAGGGAGAGTTCAATACCTCTGTAGCTGGATCTATAGAGTTGGGAAGTTCTCCTGTCTGCATAAATGCGAATGGACAGGGCACATCGAAACTTAGTTTATCCCTCGGGAATCTTAAGAAGGAAGATGTGGATATTCCCACTCTTTTGAAGCTCACTAGAGGAAGGAAGTTGGACCTCAAAAACTCTTTTTTCAAGCAGTCCCCCCAAAAAAACATGACTTTTACACTCCTTAAAGAGCGAGTTTTCACCACCCACGACTGCTCCATCTCTTACACCGATCTGGAGAAGGCCAGCTGCCAAGCTGTGGTTGGTTTTCCTGAGATCATGACAAAACAGTGCATGAAAGACAGCGGTGAACTTCAGTATGGCTCTAAGACTGCATTACGCATCCCACCGGACACCGTGATGGCTTACAGTGTCATTGAAATGACCGTCGAAAGTGACGGCTACTTTGATTTGTGCGTTTTGCCCAGTGGGTTGGAATCAGATGACATTTCCCAGAATCCCCATCCCACTTTCTCAGAGGTGGATGGTCAGTGGCCACTGATCCAGGAAGGTTTCCCACTTACTACTCTAAAAAAAGCACTTGCAGATGTTCAGACTAGCTTTTGTGCACTGGCCGACTTGTCTGCTGAAAGCCGTTCCTCCATCCTTCTCCTGTTGAGGGAGATTCTGACAGACAGATCTGTCTTGTCTGCTTTGGTGGATAGACTTGAGATGTTAAGCAGCAGTGAAGCTCCGTGCTTCTTAAACAACGAGTTGTCTGAAAAACAGAGCCAGATCATTGATGCTGTTTTAGACTTACTGAAGGGTGAACAACTTGACAAGAGTGGACTTACCACATCAACGTCAATGTCCAGTTATAATGGATGTCAGGTATCAACAGCCAATCACAGTGATCCTCTTTTGGAATCTGAGTTAAATGGATCAAGCCCCGTACCAGAAGAACAGAATGGATGTCACGCAGCTGTATCCCATCAGAACGGATGCTCAACAAAAGCAAGCAGGCAGAGCATGGAGCTTATGAATGTTATGAAAATGCTGATCAATGCTCTTGAGGAACTCACGGATGCTGGACTGGACCTGCTAGAGACGTTCTGCACTCCTGAAGGTCTCCAAAGTCTACAGGATTTAGTAATTCATCTGACTACTAGCGATATGCCTATCTGCAAGGACACCATGCCTGTTTTCCTCCAAAGTGACAATGAATTCCACAGAGTAGAGGAACTTTTTAAGTCATGCAATGTTTTGCTACGGAAAGAGAATGACACATTAACCTCAGAAATTACCTGTAGAGAGGGCTTCCTCCCAATGGTTCTGTGCATTGCCATCCATGGCCTTGCATCCTTTGTTGCTGCATGACAGTGCTAGTGCTTTTACTAACAACTGAAATAGGCTGTTAAAGTGTTAAGTCATTTTAAAATGTTTTCCCAGTTACAAAGACATATTAGTTCATGCAGAAATGACTTGCTTTAAATTTAATGTTCCTGTATGCAGACATTTGCTTGCTTATTAATATTTTGCCCTAATTTTATCTTTGTTAATTCCATAAGAGTAATCAGTCTAATTGTAATATATCTTAGAGGTCTGCAAGCCCGTCGGGTCCCGACATGCTGAGTCCATTCGGCCGGGCTTGGGCCATTTTTTTTTTTGTACAGATTGGGCTTGGGCTCGGGCTCATTTAGCTTCTCTCCTTTTATCGTGCCCATATACGCGCAGAGTACACATAGGCTACTGTATGAAATACTGTTATAATGGTTATATTATAAATATTATACATCGCCTACACAATACGCAACGCATATACGCACAGCATACAGGTGACAGTTGACCATGCAGGACATCAGGGGGAAGTTGGAGCATGGGGAGTTATTAAAACTTTAAGAAATGCATCTATACGGATTATGATTATAATGAAAGAGATTTCGGTATGTTTTATTTCGTTAAGTACTTATATAGATATATTTATCATGTCTGTGAGGCATCTTTTTCACTGAGATTCAGTTCATTTTTGTGAAGCTTTCCTGTTAAAGACGAGACGGCAGAAAGCGCATCATGTTTGTTTTCTTTATTTTATAAAAGCACATTTTGTTGATATTGTGAGTGAACAAAAATAAAAGTAGACCCTTTACAGTTAAAAGGATACGGTACCGGATACATGTACCTCTAGATCATAATCGTCTACCTGCATGCATGCAATGCTGGTCGTAATCATGGTTCATGAGCTGATTTCGTGTGTCGAAATCGTAATCCGCTCACAATACGACACAGCATACGTAGCCGGGAAGCATCAAGTGTAGACGCCTTGGAGGTGACCTAAAT

> GSDMEa [*Cyprinus carpio*]

MFDKATKKLVRQIDHKGALIASSRLNDSGKLQVLAVVQKTQKGWFWQQTKYRPTGFKLNDLLEGDPIKPVCEEKEFVSFEGEFNTSVAGSIELGSSPVCINANGQGTSKLSLSLGNLKKEDVDIPTLLKLTRGRKLDLKNSFFKQSPQKNMTFTLLKERVFTTHDCSISYTDLEKASCQAVVGFPEIMTKQCMKDSGELQYGSKTALRIPPDTVMAYSVIEMTVESDGYFDLCVLPSGLESDDISQNPHPTFSEVDGQWPLIQEGFPLTTLKKALADVQTSFCALADLSAESRSSILLLLREILTDRSVLSALVDRLEMLSSSEAPCFLNNELSEKQSQIIDAVLDLLKGEQLDKSGLTTSTSMSSYNGCQVSTANHSDPLLESELNGSSPVPEEQNGCHAAVSHQNGCSTKASRQSMELMNVMKMLINALEELTDAGLDLLETFCTPEGLQSLQDLVIHLTTSDMPICKDTMPVFLQSDNEFHRVEELFKSCNVLLRKENDTLTSEITCREGFLPMVLCIAIHGLASFVAA
